# Biosynthesis of Fungal Natural Products Involving Two Separate Pathway Crosstalk

**DOI:** 10.3390/jof8030320

**Published:** 2022-03-21

**Authors:** Guangzhi Dai, Qiyao Shen, Youming Zhang, Xiaoying Bian

**Affiliations:** Helmholtz International Lab for Anti-Infectives, Shandong University-Helmholtz Institute of Biotechnology, State Key Laboratory of Microbial Technology, Shandong University, Qingdao 266237, China; daiguangzhi@sdu.edu.cn (G.D.); syndeeyao@foxmail.com (Q.S.); zhangyouming@sdu.edu.cn (Y.Z.)

**Keywords:** biosynthetic pathway crosstalk, natural product, biosynthetic gene cluster, polyketide, fungi, bioactivity

## Abstract

Fungal natural products (NPs) usually possess complicated structures, exhibit satisfactory bioactivities, and are an outstanding source of drug leads, such as the cholesterol-lowering drug lovastatin and the immunosuppressive drug mycophenolic acid. The fungal NPs biosynthetic genes are always arranged within one single biosynthetic gene cluster (BGC). However, a rare but fascinating phenomenon that a crosstalk between two separate BGCs is indispensable to some fungal dimeric NPs biosynthesis has attracted increasing attention. The hybridization of two separate BGCs not only increases the structural complexity and chemical diversity of fungal NPs, but also expands the scope of bioactivities. More importantly, the underlying mechanism for this hybridization process is poorly understood and needs further exploration, especially the determination of BGCs for each building block construction and the identification of enzyme(s) catalyzing the two biosynthetic precursors coupling processes such as Diels–Alder cycloaddition and Michael addition. In this review, we summarized the fungal NPs produced by functional crosstalk of two discrete BGCs, and highlighted their biosynthetic processes, which might shed new light on genome mining for fungal NPs with unprecedented frameworks, and provide valuable insights into the investigation of mysterious biosynthetic mechanisms of fungal dimeric NPs which are constructed by collaboration of two separate BGCs.

## 1. Introduction

Fungi produce a plethora of chemically diverse natural products (NPs) with various bioactivities and a wide variety of applications in medicine and agriculture [1]. Because of this, fungal NPs have been historically recognized as an invaluable source of inspiration for the development of drug leads for the treatment of infections and cancer, as well as the prevention of crop damage, such as lovastatin (the cholesterol-lowering drug), mycophenolic acid (the immunosuppressive drug), and pyripyropene A (the insecticide) [2,3,4]. In addition, fungal NPs with novel molecular scaffolds provide excellent templates for the chemical synthesis of new bioactive compounds [5]. 

In fungi, the natural products are commonly synthesized by the genes arranged in a contiguous fashion as a biosynthetic gene cluster (BGC) [6]. The core biosynthetic enzymes encoded by the BGCs mainly include polyketide synthases (PKSs), non-ribosomal peptide synthetases (NRPSs), terpene synthases, and ribosomally synthesized and post-translationally modified peptide (RiPP) biosynthetic enzymes to enlarge the variety of carbon skeletons of the products. Tailoring enzymes such as the monooxygenase, hydroxylase, and methyltransferase further increase the diversity and complexity of the molecules [7]. With the development of genome sequencing technology, bioinformatic methods, genome mining algorithms, and scalable expression platforms, the genomic-driven approaches have revolutionized NPs discovery, greatly expand the access to the chemical repertoire of fungal-derived NPs, and provide unprecedented opportunities to investigate their biosynthetic mechanisms [8]. Several vital factors that regulate the expression of fungal BGCs, including the environmental signals, transcriptional regulation, and epigenetic regulation, have been summarized by Keller [9], which might be instructive for genome mining and activation of the silent BGCs.

In most reported studies, the microbial NPs biosynthetic pathways are generally completed by one single BGC. However, in some interesting but rare cases, the products have been demonstrated to be constructed via the hybridization of two precursors, which are biosynthesized by two separate BGCs. For instance, the antithrombotic myxadazoles, a family of novel chimeric compounds isolated from *Myxococcus* sp. SDU36, consists of N-ribityl 5,6-dimethylbenzimidazole and a linear fatty acid chain endowed with an isoxazole ring. Interestingly, a non-canonical PKS/NRPS biosynthetic pathway and the vitamin B12 metabolism pathway were proved to interwind together through the construction of isoxazole-benzimidazole hybrids [10]. Tasikamides, recently isolated from *Streptomyces tasikensis* P46, contain a rare hydrazone group that joins the cyclic peptide scaffold to an alkyl 5-hydroxylanthranilate (AHA) moiety. This research also addressed that the biosynthesis of tasikamides required the coupling of two separate gene clusters, with one BGC encoding a NRPS pathway for assembling the cyclic peptide scaffold, and the other BGC encoding the AHA-synthesizing pathway [11]. These works illustrate that functional crosstalk between two different biosynthetic pathways is not only of considerable value in increasing the structural diversity of NPs, but also a more effective way to construct new drug leads of natural origin. 

Several key issues need to be considered when we investigate the biosynthetic mechanism of two separate BGC co-participation in NP biosynthesis: (a) identification of each BGC and characterization of the necessary gene function; (b) isolation of sufficient quantity of key intermediates through gene knockout, enzymatic catalyzation, and heterologous expression; and (c) determination of whether the coupling process of the two separate BGCs is spontaneous or enzymatic. Thus, in-depth understanding of the biosynthetic mechanism for the convergence of two distinct biosynthetic pathways will provide an alternative to accelerate the discovery of NPs with novel skeletons, as well as shed new light on which enzyme(s) could catalyze the formation of C-C, C-N, or N-N bonds that link two biosynthetic precursors together. In this mini-review, we summarized some fungal NPs with novel skeletons produced by the crosstalk between two discrete biosynthetic gene clusters, mainly including polyketides, meroterpenoids, and non-ribosomal peptides. We highlighted their biosynthetic processes, which might provide valuable insights into the coupling mechanism of two separate BGCs in fungal NP biosynthesis.

## 2. Fungal Polyketide Biosynthesis Involving Two Separate Pathway Crosstalk

### 2.1. The Biosynthesis of Penilactones A and B 

**Figure 1:**
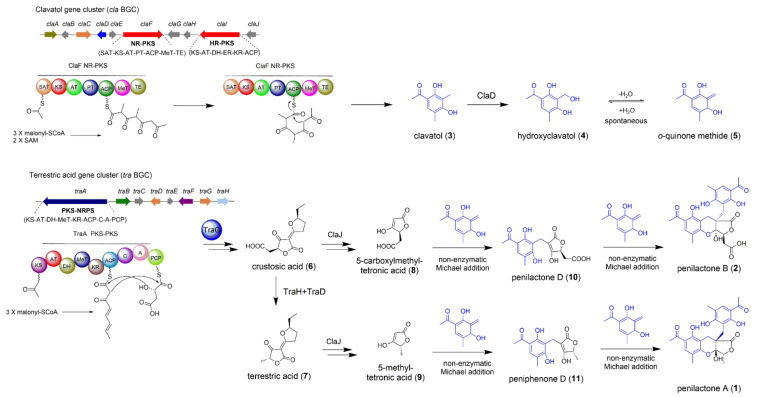
The biosynthetic pathway of fungal polyketides penilactones A (**1**) and B (**2**). The Michael addition that triggers the coupling of *cla* and *tra* BGCs is nonenzymatic.

The representative examples about non-enzymatic Michael addition mediated the coupling process of polyketide–polyketide hybrids were highly oxygenated fungal polyketides penilactones A (**1**) and B (**2**), which were firstly isolated from an Antarctic deep-sea derived fungus *Penicillium crustosum* PRB-2 by Li and co-workers [12]. The biosynthetic pathway was proposed to be originated from the hybridization of one *o*-quinone methide unit (**5**) and a γ-butyrolactone moiety through 1,4-Michael addition to complete the carbon skeleton construction of **1** and **2**. A biomimetic total synthesis was subsequently achieved to confirm the biosynthetic hypothesis [13]. Considering the enzymes for Michael addition involved in **1** and **2** biosynthesis have not been reported yet, Li and co-workers identified two separate BGCs (termed as *cla* and *tra* BGCs in this review) responsible for the biosynthesis of **1** and **2** through the gene deletion and heterologous expression in *Aspergillus nidulans* [14]. The core non-reducing polyketide synthase (NR-PKS) ClaF in the *cla* BGC is responsible for the biosynthesis of crucial intermediate clavatol (**3**) (Figure 1). The nonheme Fe^II^/2-oxoglutarate-dependent oxygenase ClaD oxidized **3** into hydroxyclavatol (**4**), which spontaneously underwent dehydration into the crucial intermediate *o*-quinone methide (**5**). For *tra* BGC, a hybrid polyketide synthase-nonribosomal peptide synthetase (PKS-NRPS) TraA and a *trans*-acting enoyl reductase (ER) TraG collaboratively catalyzed the formation of crustosic acid (**6**) precursor. The nonheme Fe^II^/2-oxoglutarate-dependent oxygenase TraH subsequently catalyzed the oxidative decarboxylation of **6** into dehydroterrestric acid, the terminal double-bond of which was finally reduced by the flavin-dependent oxidoreductase TraD into an important intermediate terrestric acid (**7**) [15]. The results of precursors feeding experiments in *ΔtraA* mutant confirmed that both **6** and **7** were the on-pathway intermediates, and could be transformed into 5-carboxylmethyltetronic acid (**8**) and 5-methyltetronic acid (**9**), respectively. The fascinating issue associated with the biosynthesis of **1** and **2** is which enzyme(s) could catalyze the Michael addition to couple two building blocks together. Surprisingly, incubation of **8** with **4** in water at 25 °C led to the formation of penilactone D (**10**) as the major product, and **2** as the minor product (Figure 1). Incubation of **9** with **4** generated peniphenone D (**11**) as the major product as well as **1** as the minor product. Both **10** and **11** were separately incubated with **4**, the formation of **1** and **2** could be observed (Figure 1). These results unambiguously indicated that the Michael addition involving in the biosynthesis of **1** and **2** is nonenzymatic and could happen spontaneously.

In general, the combination of enzymatic and nonenzymatic reactions originated from the crosstalk between two separate biosynthetic pathways significantly enriched the structural diversity of fungal NPs. The biosynthesis of fungal polyketides penilactones A and B provides an excellent example to investigate that how two different BGCs interwind at a gene cluster level.

### 2.2. The Biosynthesis of Dalmanol A and Acetodalmanol A 

**Figure 2:**
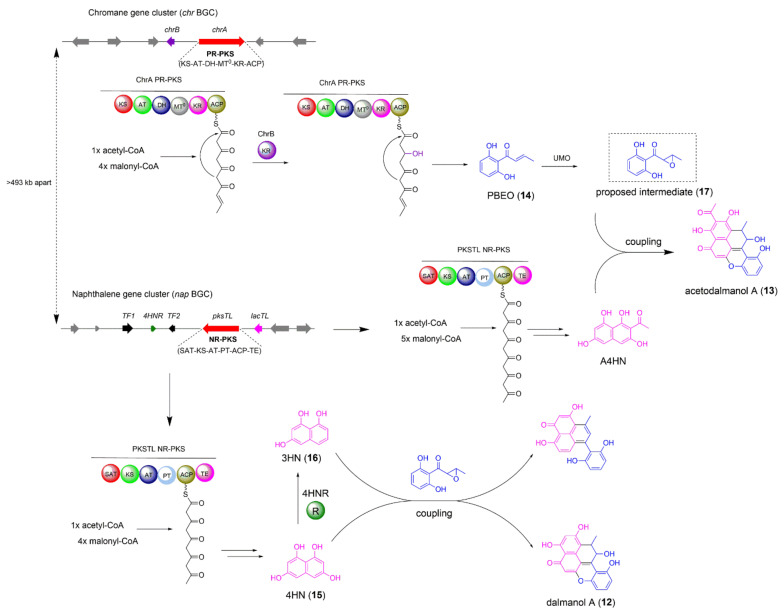
The biosynthetic pathway of fungal polyketides dalmanol A (**12**) and acetodalmanol A (**13**).

The mantis-associated fungus *Daldinia eschscholzii* TL01 is known to produce novel polyketides including dalmanol A (**12**) and acetodalmanol A (**13**) with immunosuppressive bioactivity [16,17,18]. The structural characteristic of **12** and **13** implied that their carbon skeletons construction involved in co-participation of two building blocks naphthalene and chromane. Tan and co-workers conducted the pioneering work to identify two separate BGCs (termed as *chr* and *nap* BGCs in this review) by gene deletion as well as heterologous expression [19]. The *chr* BGC is responsible for chromane biosynthesis and the *nap* BGC biosynthesizes naphthalene. This work addressed the two-gene-cluster crosstalk based biosynthetic pathways of dalmanol A and acetodalmanol A (Figure 2).

In plants and fungi, the assembly of chromane-based aromatic polyketides have been reported to be biosynthesized by both type III PKS and partially reducing type I PKS (PR-PKS) [20,21]. The results of the quantitative reverse transcription PCR analysis, targeted gene deletion, and heterologous expression experiments supported that the PR-PKS ChrA and the keto-reductase ChrB are indeed responsible for the formation of vital intermediate 1-(2,6-dihydroxyphenyl)but-2-en-1-one (PBEO,**14**) (Figure 2). Thus, the genes encoding PR-PKS ChrA, the keto-reductase ChrB, and a transporter constitute the *chr* BGC, which is located on Scaffold_36 in the genome (Figure 2). The NR-PKS gene *pksTL* in *Daldinia eschscholzii* TL01 has been confirmed to be responsible for the biosynthesis of naphthalene-based polyketide 1,3,6,8-tetrahydroxynaphthalene (4HN, **15**) by gene deletion [22]. Co-expression of the NR-PKS pksTL and 4HN reductase 4HNR in *A*. *oryzae* led to the accumulation of 1,3,6-tetrahydroxynaphthalene (3HN, **16**), a biosynthetic precursor for the assembly of dalesconols, which are polyketides also isolated from *Daldinia eschscholzii* TL01. The genes encoding the NR-PKS pksTL, 4HN reductase 4HNR, two transcription factors, and a laccase constitute the *nap* BGC, which is within Scaffold_20 in the genome and locates at least 493 kb away from the *chr* BGC. Only when *pksTL*, *chrA*, and *chrB* were co-introduced into *A*. *oryzae*, the polyketides 12 and 13 could be successfully produced. The critical biosynthetic process for the coupling of *chr* and *nap* BGCs was proposed to be the epoxidation of PBEO (14) to the proposed intermediate 17 (Figure 2). The results of enzymatic activity inhibition experiments revealed an unspecific P450 monooxygenase located elsewhere in the genome of *A*. *oryzae* host might be responsible for the epoxidation of 14 to 17, which triggered the cross-cluster coupling of both *chr* and *nap* BGCs. 

Overall, this work further illustrated that two separated BGCs crosstalk is a promising access to improve the structural diversity of fungal NPs. Understanding the regulatory mechanism of the multiple-gene-cluster coupling is of great significance in establishing the synthetic biology approaches to discover NPs with novel skeletons and potential biological activities.

### 2.3. The Biosynthesis of Azasperpyranone A

**Figure 3:**
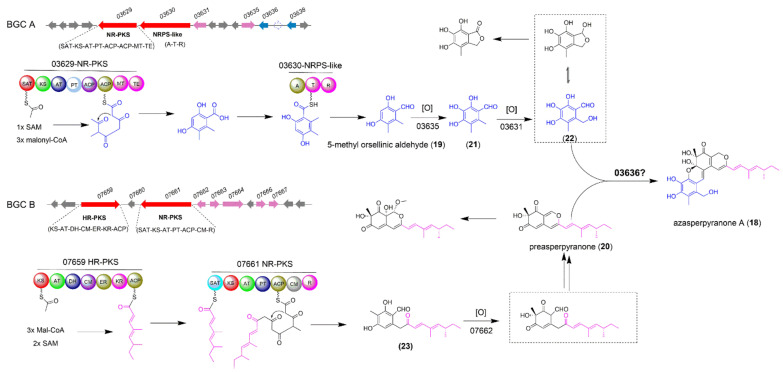
The biosynthetic pathway of fungal polyketide azasperpyranone A (**18**).

Azaphilones, a group of structurally related fungal polyketides, contain a highly oxygenated bicyclic pyrone quinone moiety, and exhibit a broad range of bioactivities including anticancer, antifungal, and antiviral activities [23]. Azasperpyranone A (**18**), recently isolated from *A*. *terreus*, contains a highly oxygenated pyranoquinone moiety possessing a 6/6/6/6 tetracyclic ring system, and shows potential anticancer activity [24]. Scrutiny of the structural feature of azasperpyranone An (**18**) revealed that two building blocks 5-methyl orsellinic aldehyde (**19**) and preasperpyranone (**20**) are the biosynthetic precursors. Lu and co-workers have identified two separate BGCs by gene deletion, including the BGC A responsible for polyhydric phenol formation and the BGC B involving the azaphilonoid scaffold construction (Figure 3) [24]. 

In a previous study, the full-length, intron-free open reading frames of two core genes *ATEG*_*03629* and *ATEG*_*03630* from BGC A, which encode a NR-PKS and a NRPS-like enzyme, respectively, were co-transformed into *Saccharomyces cerevisiae* to produce the intermediate **19** (Figure 3) [25]. Then the FAD-dependent monooxygenase (FMO) encoded by *ATEG*_*03635* gene catalyzed the hydroxylation of **19** to afford the intermediate **21**, which was subsequently oxidized into the crucial precursor **22** by the P450 monooxygenase encoded by *ATEG*_*03631* gene. In BGC B, the two core genes *ATEG*_*07659* encoding a highly reducing PKS (HR-PKS) and *ATEG*_*07661* encoding a NR-PKS were heterologously co-expressed in *A*. *nidulans* to produce **23** [26,27]. Compound **23** was rapidly converted into an important precursor preasperpyranone **20** by the FMO encoded by gene *ATEG*_*07662*. The remaining gap for the entire biosynthetic pathway of azasperpyranone A is that which enzyme could couple the two vital intermediates **20** and **22** together by catalyzing the formation of C-C and C-O bonds. Deletion of the gene *ATEG_03636* with unknown function abolished the production of **18**, but accumulated two precursors **19** and **20**, which implied the formation of **18** was more likely catalyzed by ATEG_03636 rather than caused by the spontaneous reaction between **20** and **22** (Figure 3). The authors also found that the ATEG_07667 in BGC B could indirectly regulate the cluster-specific regulators ATEG_03638 in BGC A and ATEG_07666 in BGC B to collaboratively synthesize the anti-cancer compound **18**. This interesting collaborative model in the fungal NPs biosynthesis provides new clues for the investigation of regulatory mechanism for other novel natural products which were biosynthesized by two separate BGCs crosstalk. 

## 3. Fungal Meroterpenoids Biosynthesis Involving Two Separate Pathway Crosstalk

### The Biosynthesis of Austinol

**Figure 4:**
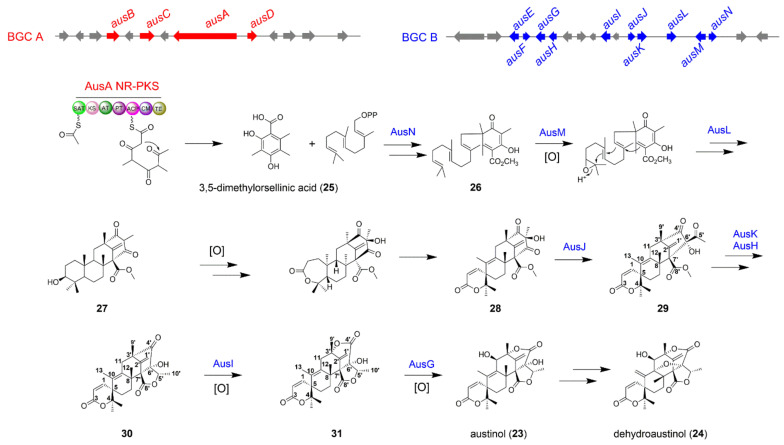
The biosynthetic pathway of fungal meroterpenoids austinol (**23**) and dehydroaustinol (**24**) in *A*. *nidulans* LO2026.

Meroterpenoids are an important class of fungal NPs [28], some of them have been developed as the drug candidate for treatment of Alzheimer’s disease such as territrem [29], and the drug lead for insecticide such as pyripyropene A [30]. Fungal meroterpenoids are usually biosynthesized by a single BGC which encodes a polyketide synthase, a terpene cyclase, a prenyltransferase, and other essential tailoring enzymes to produce polyketide and terpenoid precursors [31]. Deletion of *sumO* gene, encoding the small ubiquitin-like protein SUMO, significantly altered the profiles of secondary metabolites in *A*. *nidulans*. Austinol (**23**) and dehydroaustinol (24) were identified from the *ΔsumO* mutant [32]. Nielsen et al. first reported the NR-PKS AusA responsible for the formation of polyketide precursor 3,5-dimethylorsellinic acid (DMOA) (**25**) in austinol biosynthesis [33]. This conclusion was also verified by Wang et al. [34]. Interestingly, Wang and co-workers found that no prenyltransferase gene was located near NR-PKS gene *ausA*, which suggested that the genes responsible for the biosynthesis of **23** and **24** might be separated in the genome of *A*. *nidulans* LO2026 [34]. Using the UbiA sequence as a query to blast the prenyltransferase homologs in the genome of *A*. *nidulans*, Wang et al. found the top two candidate genes *AN9259.4* (designated as *ausN*, on chromosome VIII) and *AN8142.4*. Deletion of *ausN* abolished the production of **23** and **24**, and accumulated the polyketide precursor **25**. By a set of gene deletions around *ausA* and *ausN*, they identified the two separate BGCs: the BGC A containing the necessary genes *ausA*-*D* and the BGC B consisting of the biosynthetic genes *ausE*-*N*. The biosynthetic pathway for **23** and **24** was also proposed (Figure 4). Firstly, the polyketide synthase AusA is responsible for the formation of **25**, then the aromatic prenyltransferase AusN catalyzed the C-alkylation of **25** using farnesyl pyrophosphate to form intermediate **26**. The epoxidase AusM catalyzed the epoxidation of the prenylated polyketide intermediate **26**, followed by cyclization catalyzed by a terpene cyclase AusL to form the tetracyclic intermediate **27**. The formation mechanism of the lactone system and spiro-ring in compound **28** has been investigated by Abe group [35]. The co-operation of the non-heme iron-dependent dioxygenase AusE, the hydroxylase AusB, and the Baeyer–Villiger monooxygenase AusC transformed the substrate **27** into **28** [35]; The hypothetical protein AusJ might be responsible for the acid-catalyzed keto-rearrangement and ring contraction of the tetraketide portion in intermediate **28** to generate intermediate **29**. The authors speculated that the AusK is responsible for reducing the C-5′ keto of **29** to hydroxyl group, and the hypothetical protein AusH might function as an accessory enzyme collaboratively working with AusK to alter AusK stereospecificity for its product **30**. The Baeyer−Villiger monooxygenase AusI inserted an oxygen atom between the C-4′ and vicinal carbon at C-3′ of **30** to create a lactone ring in **31**. Finally, the P450 monooxygenase AusG might catalyze the C-11 hydroxylation of **31** to form final product austinol (**23**) (Figure 4). 

DMOA-derived fungal meroterpenoids possess complicated structures and attracted researchers’ attention to investigate the biosynthetic pathway. Some fungal meroterpenoids such as anditomin and andrastin have been studied in detail [36,37,38]. All of the necessary genes for anditomin or andrastin biosynthesis are clustered in one single BGC. However, the biosynthesis of austinol and dehydroaustinol in *A*. *nidulans* LO2026 needs the pathway crosstalk between BGC A and BGC B. This provides an intriguing and valuable insight that fungi could use a variety of strategies to expand the skeletal diversification and subtlety regulate the crosstalk between separate biosynthetic pathways to multiply the number of NPs produced by these BGCs. 

## 4. Fungal Non-Ribosomal Peptide Biosynthesis Involving Two Separate Pathway Crosstalk

### 4.1. The Biosynthesis of Spirotryprostatin A

**Figure 5:**
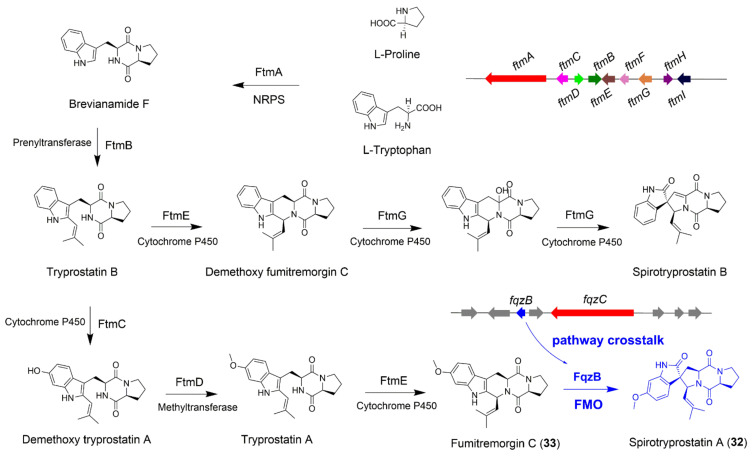
The biosynthetic pathway of fungal non-ribosomal peptide spirotryprostatin A (**32**). The FMO FqzB from the *fqz* BGC catalyzes the formation of spiro-carbon in spirotryprostatin A.

Many NPs bearing the spiro-carbon system exhibit potential biological activities. Intrigued by the privileged structure and usefulness of spiro carbon system, a great deal of attention has been paid to their catalytic enantioselective synthesis [39,40]. Understanding the mechanism of spiro-carbon biosynthesis and identifying the versatility of enzymes responsible for the spiro-carbon formation are also of great significance. Spirotryprostatins belong to non-ribosomal peptides that isolated from *A*. *fumigatus*, and known for their pharmaceutical importance and application in cancer treatment [41]. The formation of the spiro-ring moiety in spirotryprostatins remained unknown and aroused Watanabe and co-workers’ interest to solve this mystery [42]. The authors utilized *S*. *cerevisiae* and *A*. *niger* as the heterologous hosts to efficiently express the whole biosynthetic pathways of spirotryprostatins, and obtained crucial intermediates to identify two pathways for spiro-carbon formation, namely an epoxide route catalyzed by the FMO FqzB and a radical route catalyzed by the cytochrome P450 FtmG (Figure 5) [42]. Spirotryprostatins possess the diketopiperazine frameworks, and show the structural similarity to fumitremorgins and fumiquinazolines, suggesting the peptide backbone of these compounds could be biosynthesized by the NRPS using L-proline and L-tryptophan as the biosynthetic precursors [43,44]. When *ftmA*-*E* five genes were expressed in *A*. *niger*, no intermediates featuring the spiro-carbon were isolated, implying other indispensable genes are needed to be introduced. Based on the prior researches about the biosynthetic mechanism of spiro-carbon formation [45,46,47], the authors creatively introduced the FMO FqzB-encoding gene *fqzB*, which is located within the fumiquinazoline BGC (designed as fqz BGC in this review), into the *A*. *niger*/ *ftmA*-*E* mutant, and successfully isolated pirotryprostatin A (**32**) (Figure 5). In vitro enzymatic assay revealed that FqzB could transform fumitremorgin C (**33**) into **32** by epoxidation mediated semipinacol-type rearrangement (Figure 5). These fascinating results not only emphasize the important function of FMOs as the intersection to trigger the coupling of two separate BGCs, but also highlight that the crosstalk between different biosynthetic pathways allows the structural diversification in NP biosynthesis.

### 4.2. The Biosynthesis of Echinocandin B 

**Figure 6:**
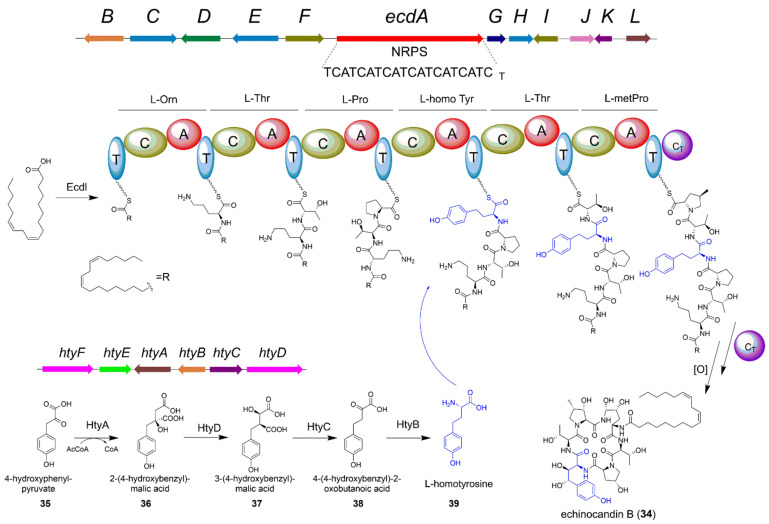
The biosynthetic pathway of fungal non-ribosomal peptide echinocandin B (**34**). The separate *hty* BGC is responsible for the L-homotyrosine moiety formation.

Echinocandins, a family of fungal lipohexapeptides, are firstly isolated from *Emericella rugulosa* NRRL 11440, and exhibit excellent antifungal activities to the opportunistic pathogenic *Candida* strains. Structural modifications of echinocandin B (**34**), especially for the fatty acid moiety, successfully led to the generation of FDA-approved drug anidulafungin, which is a semisynthetic derivative of **34** and contains a substituted terphenyl acyl chain. To better understand how microbes use simple precursors to synthesize complex NPs, Tang and co-workers performed the groundbreaking work to identify and characterize the BGC of echinocandin B [48]. Four nonproteinogenic amino acids including 4*R*,5*R*-dihydroxyl-L-ornithine, 3*S*-hydroxyl-4*S*-methyl-L-proline, 4*R*-hydroxyl-L-proline, and 3*S*,4*S*-dihydroxyl-L-homotyrosine, as well as a long chain fatty acyl amide were contained in echinocandin B (Figure 6). These unusual structural units implied an interesting biosynthetic mechanism of echinocandin B.

By bioinformatics analysis, the NRPS EcdA containing six modules was identified. Gene deletion of *ecdA* confirmed its vital role in the peptide backbone formation of echinocandin B. Other biosynthetic genes, such as *ecdI* encoding a fatty-acyl-AMP ligase (EcdI), *ecdG* and *ecdK* encoding two α-ketoglutarate dependent oxygenases, and *ecdH* encoding a heme-iron-dependent cytochrome P450 oxygenase, were all in proximity to *ecdA* to constitute the *ecd* BGC. However, the genes responsible for the biosynthesis of L-homotyrosine were not present in the vicinity of *ecd* BGC, indicating a separate BGC should reside elsewhere in the genome. The putative 2-(4-hydroxybenzyl)-malic acid (**36**) is proposed to be the biosynthetic intermediate of L-homotyrosine (**39**) (Figure 6). Considering the isopropyl-malate synthase (IPMS) is reported to catalyze the condensation of α-ketovalerate with acetyl-CoA in the leucine biosynthesis, one IPMS homology in *E*. *rugulosa* genome was proposed to catalyze the condensation of 4-hydroxyphenyl-pyruvate and acetate to form **36**. Using the IPMS gene from *Mycobacterium tuberculosis* as BLAST query, the authors successfully identified the *hty* BGC, which is about 42.5 kb away from the *ecd* BGC, encoding four enzymes for de novo generation of the special building block L-homotyrosine (Figure 6) [48]. The isopropyl malate dehydrogenase HtyA catalyzed aldol-type condensation of 4-hydroxyphenyl-pyruvate (**35**) and acetyl-CoA to form **36**. Then, the aconitase homology HtyD executed the isomerization of 36 to 3-(4-hydroxybenzyl)-malic acid (**37**). Thereafter, **37** underwent decarboxylation and oxidation to form 2-oxo-4-(4-hydroxybenzyl) butanoic acid (**38**) by isopropyl malate dehydrogenase homologue HtyC. Finally, the transaminase HtyB catalyzed the transamination of **38** to form **39**. In general, the biosynthesis of echinocandin B needs the coupling of two sperate BGCs, the *ecd* and *hty* BGCs. The *hty* BGC provides an important biosynthetic precursor L-homotyrosine which was recognized by the fourth A domain of the NRPS EcdA. Understanding the biosynthetic mechanism of echinocandin B will facilitate us to take advantage of synthetic biology techniques to bioengineer NRPSs to generate bioactive compounds [49,50,51]. 

## 5. Representative Fungal NPs Might Be Biosynthesized by Two Separate Pathways Crosstalk 

### 5.1. Delitschiapyrone A

**Figure 7:**
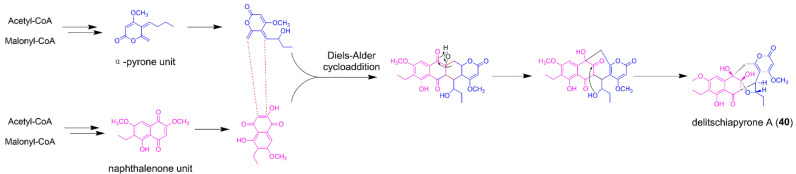
The proposed biosynthetic pathway of delitschiapyrone A (**40**).

Delitschiapyrone A (**40**) is a fungal polyketide bearing an unprecedented 6/6/7/5/6-fused pentacyclic ring system (Figure 7), and isolated from a solid culture of the leaf-associated fungus *Delitschia* sp. FL1581 [52]. The absolute configuration of **40** was determined by spectroscopic analysis, X-ray crystallography data, and experimental and calculated ECD. A naphthalenone unit and an α-pyrone moiety were proposed to be linked together via the Diels–Alder addition followed by an α-ketol-type rearrangement to forge the pentacylic ring system of **40** (Figure 7), which suggested the biosynthetic pathway of **40** might be concerned with the crosstalk between two separate BGCs (one for naphthalenone biosynthesis and the other for α-pyrone biosynthesis). A bioinspired total synthesis of delitschiapyrone A has been achieved by simply stirring a heterogeneous mixture of two Diels−Alder substrates, which gave a hint that the intermolecular Diels−Alder reaction might be spontaneous. Recently, Houk and co-workers investigated the mechanisms and dynamics of biosynthetic formation of **40** by density functional theory (DFT) calculations and quasiclassical molecular dynamics simulations with DFT and xTB, and drew a conclusion that **40** is not formed from the proposed Diels−Alder/α-ketol rearrangement cascade but instead formed directly from a single cycloaddition reaction [53], which is of great significance to the subsequent study on the biosynthetic pathway of **40** through gene deletion, heterologous expression, and enzymatic assays. 

### 5.2. Herpotrichone A

**Figure 8:**
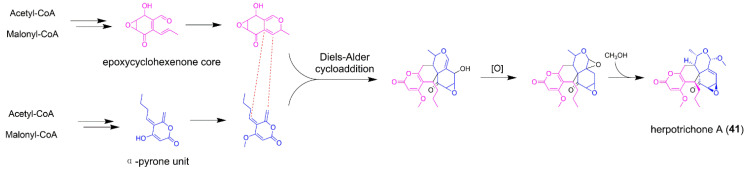
The proposed biosynthetic pathway of herpotrichone A (**41**).

Herpotrichone A (**41**) is a fungal polyketide isolated from the isopod-associated fungus *Herpotrichia* sp. SF09 with an unprecedented pentacyclic 6/6/6/6/3 skeleton, and shows outstanding anti-neuro inflammatory activities in lipopolysaccharide (LPS)-induced BV-2 microglial cells (Figure 8) [54]. Interestingly, compound **41** is also an intermolecular [4 + 2] adduct that involves the coupling of epoxycyclohexenone and α-pyrone two building blocks via the Diels–Alder addition. Recently, the biosynthetic pathways of epoxycyclohexenone-derived fungal polyketides trichoxide, sordarial, and flavoglaucin have been investigated in detail, which provide new insights into the biosynthesis of the naphthalenone unit in **41** [55,56,57]. The biosynthesis of some fungal α-pyrone-linked NPs such as alternapyrones and citreoviridin have been reported. The α-pyrone moiety in alternapyrones and citreoviridin is indeed formed by the spontaneous intramolecular cyclization of PKS AlpA and CtvA, respectively [58,59]. Thus, herpotrichone A might share the similar strategy to forge the α-pyrone unit by utilization of an unidentified PKS. Overall, fungal NPs with intermolecular Diels–Alder addition features usually have novel carbon skeletons. The unexpected architectures of these compounds may open an interesting topic such as the characterization of two separate BGCs crosstalk, and discovery of more fungal intermolecular Diels–Alderases. 

### 5.3. Citrifuran A 

**Figure 9:**
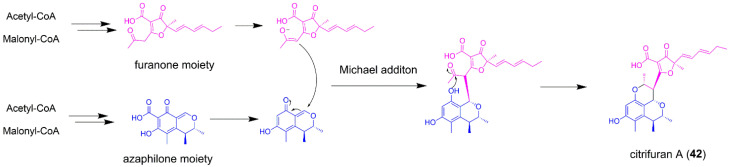
The proposed biosynthetic pathway of citrifuran A (**42**).

Citrifuran A (**42**) is produced by the centipede intestine-associated *Aspergillus* sp. through solid fermentation (Figure 7), and showed moderate inhibitory activities against LPS-induced NO production in RAW 264.7 macrophages [60]. The novel skeleton of citrifuran A was constructed by coupling of azaphilone and furanone moieties via Michael addition (Figure 9). It is obvious that the two separate BGCs’ crosstalk is indispensable to the biosynthesis of **42**. The furanone moiety was also contained in fungal polyketide gregatin A. For gregatin A biosynthesis, a single PKS GrgA with the aid of a *trans*-acting enoylreductase GrgB could biosynthesize the C_4_ and C_11_ carbon chains. More interestingly, a predicted hydrolase GrgF is responsible for the fusion two carbon chains to produce the furanone skeleton of gregatin A [61]. This unusual chain-fusing reaction might be also suitable for the biosynthesis of furanone scaffold in **42** (Figure 9). The fungal polyketide-derived mycotoxin citrinin also possesses azaphilone building block. The individual biosynthetic steps of citrinin have been studied by a combination of targeted gene knockout and heterologous gene expression in *A*. *oryzae* [62], which might provide new clues for investigation of the biosynthetic mechanism of **42**. Though the biosynthetic pathways for azaphilone and furanone have been investigated, the enzyme for catalyzation of Michael addition has not yet been identified, and needs further exploration. 

### 5.4. Acautalide A

**Figure 10:**
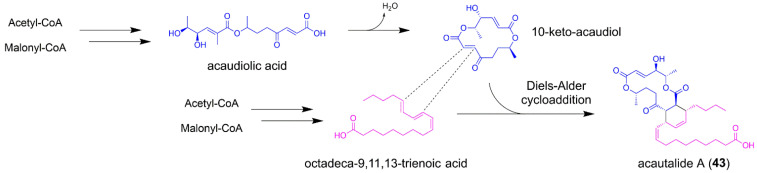
The proposed biosynthetic pathway of acautalide A (**43**).

The fungal polyketide acautalide A (**43**) is produced from the solid-state cultivation of isopod *Armadillidium vulgare*-associated *Acaulium* sp. H-JQSF on rice medium, and exhibits neuroprotective bioactivity with antiparkinsonic potential in the 1-methyl-4-phenylpyridinium-challenged nematode model [63]. The architectural features of 43 indicated that two biosynthetic precursors 10-keto-acaudiol and octadeca-9,11,13-trienoic acid intertwined together through intermolecular Diels–Alder cycloaddition (Figure 10). The 10-keto-acaudiol unit is proposed to be an early-stage precursor in the biosynthesis of acaulide and acaulins, two fungal macrodiolides previously isolated from the *Acaulium* sp. H-JQSF [64,65]. However, the biosynthetic mechanism of 10-keto-acaudiol remains unclear. The octadeca-9,11,13-trienoic acid motif might be derived from the biosynthesis of fungal polyunsaturated fatty acids, either biosynthesized from the fungal desaturation of octadecanoic acids in rice. Interestingly, the obtained **43** was in enantiomerically pure form, thus the Diels–Alder cycloaddition might be truly enzymatic. A fungal Diels–Alderase could be expected to catalyze the intermolecular [4 + 2]-cycloaddition in the assembly line of **43** [63].

## 6. Discussion

One reason that fungi endow great potentials to produce natural products with complex structures and excellent biological activities might be ascribed to the complex metabolic regulatory networks and interaction of different biosynthetic gene clusters. Understanding how simple precursors are synthesized and assembled together to construct complex natural products in organisms may promote the development of new combinational and synthetic biology strategies to create new molecules. 

Over the course of evolution, fungi have evolved different strategies to increase the diversity of their NPs to protect themselves and acclimatize to the surrounding ecological environment [66]. For example, a great progress has been made in the discovery and identification of fungal NPs with homo-dimeric or hetero-dimeric skeletons, which effectively expand structural diversity of NPs and accelerates the occurrence of new biological activities. For fungal homodimer NPs, the building blocks are mostly biosynthesized by one single BGC, and catalyzed by the crucial enzymes including cytochrome P450 enzymes, intermolecular Diels–Alderases, and multicopper oxidases to afford the homo-dimeric skeletons, such as the rugulosin A, bisorbicillinol, and viriditoxin biosynthesis [67,68,69,70]. On the contrary, a rare but intriguing phenomenon is that two different building blocks, usually produced by two separate BGCs, were coupled together to generate fungal heterodimers NPs. However, the underlying mechanisms including how two structural units are biosynthesized, and whether the two BGCs crosstalk process is enzymatic or spontaneous are still mysterious and need further exploration. The coupling reactions between the two different building blocks are various. The Diels–Alder reaction and Michael addition reaction have been reported to splice the separate biosynthetic precursors together [14,52]. To in-depth understand the biosynthetic process of two separate BGC crosstalk, we should characterize the biosynthetic gene function in two BGCs and acquire crucial biosynthetic precursors through gene knockout and heterologous expression. With the important intermediates in hand, we can further investigate whether this hybridization process is spontaneous or enzymatic. However, the determination of which enzymes responsible for the two separate BGC crosstalk process is sometimes challenging, because the corresponding biosynthetic genes might not be located within the two gene clusters, and distributed elsewhere in the genome of targeted strain. Moreover, these enzymes may be hypothetical proteins, and it is difficult to be identified through bioinformatic analysis.

In general, more endeavors are needed to carry out the research for the discovery of fungal heterodimer NPs constructed by two building blocks, which not only provides an outstanding opportunity for investigation of the currently underestimated hidden biosynthetic crosstalk, but also facilitates the discovery of new BGCs, new regulatory mechanisms, and enzyme catalysts with novel catalytic mechanisms.

## Data Availability

Not applicable.

## Abbreviations

NP	natural product
BGC	biosynthetic gene cluster
PKS	polyketide synthase
NR-PKS	non-reducing polyketide synthase
HR-PKS	highly reducing polyketide synthase
NRPS	non-ribosomal peptide synthetases
RiPP	post-translationally modified peptide
FMO	FAD-dependent monooxygenase
ER	enoyl reductase
AHA	alkyl 5-hydroxylanthranilate
IPMS	isopropyl-malate synthase
DMOA	3,5-dimethylorsellinic acid
LPS	lipopolysaccharide
PBEO	1-(2,6-dihydroxyphenyl)but-2-en-1-one
DFT	density functional theory
